# Expression of Nav1.7 in DRG neurons extends from peripheral terminals in the skin to central preterminal branches and terminals in the dorsal horn

**DOI:** 10.1186/1744-8069-8-82

**Published:** 2012-11-07

**Authors:** Joel A Black, Noémie Frézel, Sulayman D Dib-Hajj, Stephen G Waxman

**Affiliations:** 1 Department of Neurology and Paralyzed Veterans of America Center for Neuroscience and Regeneration Research, Yale University School of Medicine, New Haven, CT, 06510, USA; 2 Rehabilitation Research Center, VA Connecticut Healthcare System, West Haven, CT, 06516, USA; 3 Neuroscience Research Center (127A) VA Connecticut Healthcare System, 950 Campbell Avenue, West Haven, CT, 06516, USA

**Keywords:** Dorsal root ganglia, Dorsal horn, Intraepidermal nerve fiber, Pain pathway, Sodium channel, Spinal cord

## Abstract

**Background:**

Sodium channel Nav1.7 has emerged as a target of considerable interest in pain research, since loss-of-function mutations in *SCN9A*, the gene that encodes Nav1.7, are associated with a syndrome of congenital insensitivity to pain, gain-of-function mutations are linked to the debiliting chronic pain conditions erythromelalgia and paroxysmal extreme pain disorder, and upregulated expression of Nav1.7 accompanies pain in diabetes and inflammation. Since Nav1.7 has been implicated as playing a critical role in pain pathways, we examined by immunocytochemical methods the expression and distribution of Nav1.7 in rat dorsal root ganglia neurons, from peripheral terminals in the skin to central terminals in the spinal cord dorsal horn.

**Results:**

Nav1.7 is robustly expressed within the somata of peptidergic and non-peptidergic DRG neurons, and along the peripherally- and centrally-directed C-fibers of these cells. Nav1.7 is also expressed at nodes of Ranvier in a subpopulation of Aδ-fibers within sciatic nerve and dorsal root. The peripheral terminals of DRG neurons within skin, intraepidermal nerve fibers (IENF), exhibit robust Nav1.7 immunolabeling. The central projections of DRG neurons in the superficial lamina of spinal cord dorsal horn also display Nav1.7 immunoreactivity which extends to presynaptic terminals.

**Conclusions:**

The expression of Nav1.7 in DRG neurons extends from peripheral terminals in the skin to preterminal central branches and terminals in the dorsal horn. These data support a major contribution for Nav1.7 in pain pathways, including action potential electrogenesis, conduction along axonal trunks and depolarization/invasion of presynaptic axons. The findings presented here may be important for pharmaceutical development, where target engagement in the right compartment is essential.

## Introduction

Voltage-gated sodium channels are critical participants in neuronal excitability and transmition of electrical impulses along pain pathways, and have emerged as major targets for therapeutic intervention in pain disorders [1-4]. Of the nine sodium channel isoforms that have been cloned [5], four channels – Nav1.3, Nav1.7, Nav1.8 and Nav1.9 – have received intense scrutiny for their contributions to nociception and chronic pain disorders [3,6]. In particular, Nav1.7 has recently emerged as a target of considerable interest, since loss-of-function mutations in *SCN9A*, the gene that encodes Nav1.7, are associated with congenital insensitivity to pain [7-9] and gain-of-function mutations have been linked to pain in erythromelalgia [10-13] and paroxysmal extreme pain disorder (PEPD) [14-16]. In addition gain-of-function variants in Nav1.7 have more recently been identified in nearly 30% of patients diagnosed with painful idiopathic small fiber neuropathy, suggesting of a contribution of hyperactive Nav1.7 channels in axonal degenerative pathways and pain that accompanies neuropathies [17-19].

Nav1.7 is a tetrodotoxin-sensitive (TTX-S), fast-activating and fast-inactivating sodium channel that recovers (reprimes) slowly from fast-inactivation [20]. Nav1.7 is also characterized by slow closed-state inactivation, which allows the channel to pass sodium current (ramp current) in response to small, slow depolarizations [21-23].

Nav1.7 is preferentially expressed in dorsal root ganglia (DRG) and sympathetic neurons [13,24-27], and has recently beenshown to be the main sodium channel isoform in olfactory sensory neurons and their processes [28,29]. In DRG, Nav1.7 is expressed in A- and C-fiber type neurons, but is more prominently expressed in small diameter neurons, with 85% of functionally-identified nociceptive neurons exhibiting Nav1.7 immunolabeling [26]. While Nav1.7 has been localized to the somata of DRG neurons, descriptions of the expression and organization of Nav1.7 along peripheral and central unmyelinated and myelinated projections of these sensory cells are limited. Nav1.7 was colocalized with peripherin-positive fibers [30] in a teased nerve preparation, and it recently was demonstrated that intraepidermal nerve fibers (IENF), which are the peripherally-directed terminals of nociceptive DRG neurons, express Nav1.7 [31]. While Nav1.7 labeling has been reported in spinal cord dorsal horn [32], it has yet to be established whether Nav1.7 is expressed within the axon branches or central terminals of DRG neurons.

In the present report, the expression and distribution of Nav1.7 in unmyelinated and myelinated DRG neurons along the entire trajectory from peripheral to central terminals is described. The results demonstrate that Nav1.7 is highly-expressed in small diameter DRG and their peripherally- and centrally-directed processes from the skin to the CNS. Notably, Nav1.7 is clearly present in both peripheral axon terminals of DRG neurons and also in their centrally-directed axons within the dorsal horn, extending to central perterminal and terminal regions of these pain-signalling neurons. Moreover, Nav1.7 is robustly expressed at nodes of Ranvier in a subpopulation of small diameter myelinated fibers. These observations are consistent with critical roles for Nav1.7 channels at multiple sites within nociceptive DRG neurons and their processes.

## Results

Sodium channels play important roles in nociception and chronic pain syndromes [3,6], with a specific sodium channel isoform, Nav1.7, being identified that is critical in pain signaling [33]. To provide a molecular anatomical substrate for understanding the contribution of Nav1.7 channels to nociception and pain disorders, we examined by immunocytochemical methods the expression and distribution of Nav1.7 in dorsal root ganglion (DRG) neurons from peripheral free nerve ending terminals in the epidermis to central terminals in spinal cord dorsal horn.

**Figure 1:**
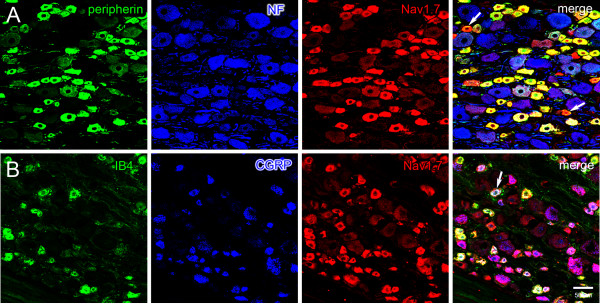
**Expression of Nav1.7 in DRG neurons. A**. DRG sections were reacted to antibodies against peripherin, neurofilament 200 (NF) and Nav1.7. Peripherin-positive (green) neurons are generally of small diameter (<30 μm) and most exhibit colocalization (yellow) with Nav1.7 (red). A few small peripherin-negative neurons display robust Nav1.7 immunolabeling (arrows). Neurofilament 200 (NF)-positive (blue) neurons are generally larger than peripherin-positive cells and most do not display colocalization with Nav1.7. A few smaller NF-positive cells exhibit Nav1.7 immunolabeling (magenta). **B**. DRG sections were reacted with IB4-488 and antibodies to CGRP and Nav1.7. Virtually all IB4-positive (green) neurons display colocalization (yellow) with Nav1.7. Similarly, nearly all CGRP-positive (blue) neurons exhibit colocalization (magenta) with Nav1.7 (red). Few DRG neurons (arrow) display colocalization of IB4, CGRP and Nav1.7.

DRG were triple-labeled with antibodies to peripherin, a C-fiber marker [34], neurofilament-200, a marker of A-fibers [35], and Nav1.7 to determine the expression of Nav1.7 in unmyelinated and myelinated sensory neurons. Peripherin-positive DRG neurons were generally of small (<30 μm) diameter and the majority of these neurons exhibited robust Nav1.7 immunolabeling (Figure 1). Approximately 63% (232/367) of peripherin-positive DRG neurons exhibited Nav1.7 immunoreactivity. A limited number of small DRG neurons displayed prominent Nav1.7 labeling but were peripherin-negative. In contrast to the pronounced co-localization of Nav1.7 and peripherin, most neurofilament-positive DRG neurons lacked detectable Nav1.7 immunolabeling (Figure 1). Of neurofilament-positive DRG neurons, about 15% (57/377) exhibited Nav1.7 immunolabeling, which was generally displayed in medium diameter (30–40 μm) neurons. The intensity of the label in the medium diameter neurons was substantially reduced compared to that displayed by peripherin-positive cells. Large (>40 μm) diameter neurofilament-positive neurons rarely exhibited Nav1.7 immunolabeling, which was of limited intensity when present. 

C-fiber DRG neurons, which are predominantly nociceptive, can be divided into subpopulations of peptidergic, which express neuropeptides as substance P and calcitonin gene-related protein (CGRP), and non-peptidergic, which bind isolectin B4 (IB4) and lack neuropeptide expression, neurons [36]. To determine the expression of Nav1.7 in peptidergic versus non-peptidergic DRG neurons, we triple-labeled DRG with IB4, CGRP and Nav1.7. Approximately equal numbers of neurons exhibited IB4 and CGRP labeling, and there was very limited co-expression of these markers in individual neurons (Figure 1). IB4-labeled DRG neurons exhibited robust Nav1.7 immunoreactivity (Figure 1). Nav1.7 immunolabeling was also displayed in CGRP-positive neurons. Quantitative analysis of the triple-labeled DRG demonstrated that similar percentages of IB4^+^ (64.8%; 187/287) and CGRP^+^ (57.8%; 158/273) DRG neurons exhibited Nav1.7 immunolabeling.

**Figure 2:**
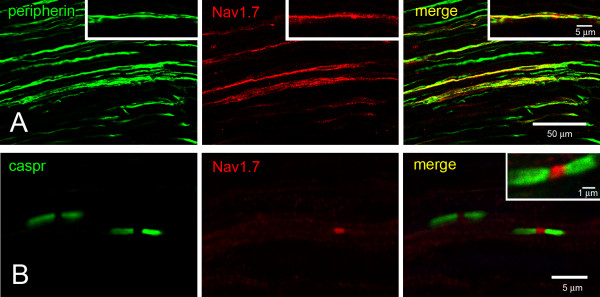
**Expresion of Nav1.7 in sciatic nerve. A**. Numerous peripherin-positive (green) unmyelinated fibers are immunolabeled in sciatic nerve and these fibers exhibit extensive colocalization (yellow) with Nav1.7 (red). Inset. At increased magnification, peripherin-positive (green) fiber displays colocalization (yellow) with Nav1.7. **B**. Nodal regions in sciatic nerve were identified by paranodal caspr (green) labeling. Nav1.7 (red) immunolabeling at a node is displayed by a small diameter (<1 μm) myelinated fiber. Not all small diameter myelinated fibers exhibit Nav1.7 labeling at nodes.

DRG neurons are psuedo-unipolar cells whose single process bifurcates, sending one branch peripherally to terminate in somatic targets and one branch centrally to terminate centrally in CNS. To determine the expression of Nav1.7 in peripherally-directed DRG fibers, sections of sciatic nerves were immunoreacted with Nav1.7, peripherin, to label unmyelinated fibers [34], and caspr (contactin-associated protein), to label paranodal regions of myelinated fibers [37]. Peripherin-positive C-fibers exhibited Nav1.7 immunolabeling (Figure 2). In favorable sections, diffuse continuous Nav1.7 labeling was observed that extended for hundreds of microns along the lengths of the C-fibers. Approximately 27% (28/104) of peripherin-positive fibers in the sciatic nerve displayed Nav1.7 immunolabeling above background levels. 

In contrast to unmyelinated fibers, myelinated fibers in sciatic nerve sections generally did not exhibit detectable Nav1.7 expression along extended lengths of the axons beneath myelin sheaths. Only ~3% (2/75) of NF^+^ axons exhibited Nav1.7 immunoreactivity along the myelinated fibers, and, when present, the immunosignal was of low intensity. In addition, Nav1.7 immunoreactivity was generally not exhibited at nodes of Ranvier. However, a subset of small diameter (<1 μm at nodes) myelinated axons displayed robust Nav1.7 labeling at their nodes (Figure 2). The Nav1.7 immunoreactivity was confined specifically to the nodal region and did not extend into the paranodal regions. Nodes of ~36% (38/107) small diameter axons displayed robust Nav1.7 labeling. Nav1.7 labeling at nodes was not detected in myelinated axons >1 μm diameter.

**Figure 3:**
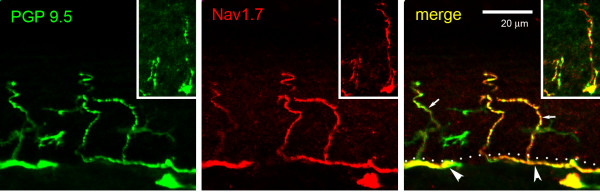
**Expression of Nav1.7 in glabrous skin. ** PGP9.5 (green) intraepidermal nerve fibers (IENF) branch from nerve bundles (arrowheads) at dermis/epidermis boundary (dotted line) and ascend in the epidermis. PGP9.5-positive IENF exhibit Nav1.7 (red) immunolabeling. Inset. Both IENF extending to stratum granulosum (more superficial) and to the stratum spinosum exhibit Nav1.7 labeling.

Within the skin, intraepidermal nerve fibers (IENF) branch perpendicularly from bundles of C- and Aδ-fibers that run parallel and subjacent to the dermis/epidermis boundary and ascend within layers of the epidermis. Peptidergic fibers generally terminate in the stratum spinosum layer, while non-peptidergic fibers terminate in stratum granulosum [38]. Subepidermal nerve bundles exhibited PGP9.5 labeling and were strongly Nav1.7 immunolabeled (Figure 3). As demonstrated in Figure 3, Nav1.7 was expressed in the free nerve terminals of both peptidergic and non-peptidergic fibers within the epidermis. Importantly, Nav1.7 immunoreactivity in IENF extended from the point of branching from the dermal nerve bundles to the terminal tips of the fibers. 

**Figure 4:**
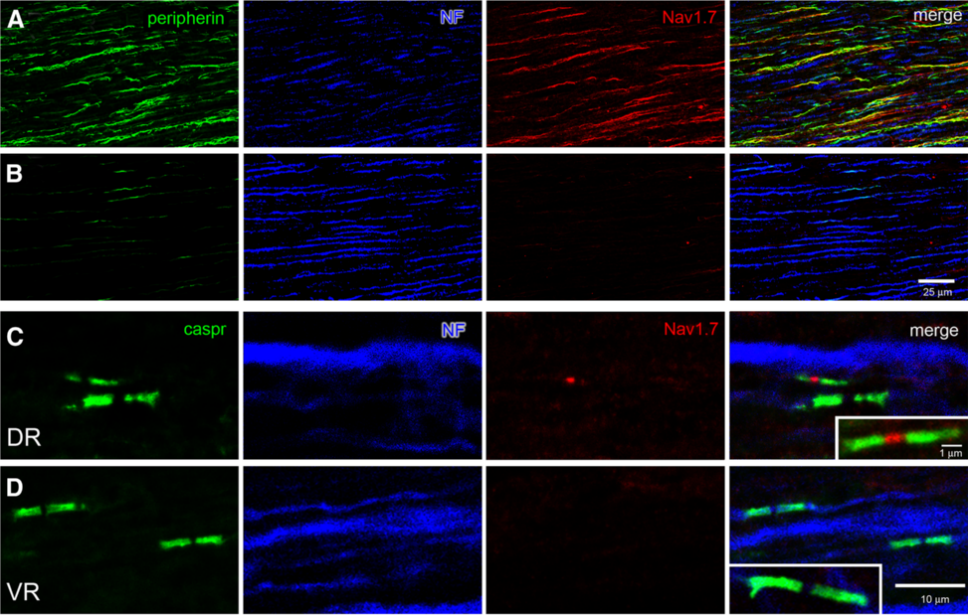
**Expression of Nav1.7 in dorsal and ventral roots. A., B.** Sections of dorsal (**A**) and ventral (**B**) roots were reacted with antibodies to peripherin, neurofilament 200 (NF) and Nav1.7. Numerous peripherin-positive (green) fibers are present in dorsal root but few in ventral root. There is extensive colocalization (yellow) of peripherin (green) and Nav1.7 in dorsal root fibers. Ventral roots do not exhibit detectable Nav1.7 labeling. **C., D.** Both dorsal (**C**) and ventral (**D**) roots display caspr paranodal labeling (green). Nav1.7 (red) immunolabeling is displayed by small diameter fiber in dorsal root but not in ventral root. Inset. Increased magnification of nodal region demonstrates that Nav1.7 labeling is confined to node.

The central processes of DRG neurons form dorsal roots along their projection to synaptic terminations in the spinal cord. Dorsal roots exhibited extensive peripherin labeling of unmyelinated sensory fibers, as well as neurofilament-positive myelinated fibers (Figure 4). Peripherin-positive fibers in dorsal roots displayed robust Nav1.7 immunolabeling along their lengths (Figure 4). The Nav1.7 labeling of peripherin-positive fibers was not focal, but could extend continuously for hundreds of microns. Similar to sciatic nerve, approximately 30% (54/185) peripherin-positive fibers in dorsal roots exhibited Nav1.7 immunoreactivity. In contrast to the Nav1.7 labeling of dorsal roots, ventral roots, which are composed primarily of peripherally-directed, neurofilament-positive axons of ventral motor neurons, did not display detectable Nav1.7 labeling.

To determine whether small diameter myelinated fibers in dorsal roots display Nav1.7 immunolabeling at nodes, similar to that observed in sciatic nerves, dorsal root sections were immunoreacted with Nav1.7 and caspr antibodies. As in sciatic nerve, a subset of small diameter (<1 μm) myelinated fibers exhibited nodal Nav1.7 immunolabeling (Figure 4). The nodal labeling was focal and was not observed in paranodal or juxtaparanodal regions, similar to that observed in sciatic nerves. Approximately 34% (20/67) of the small diameter myelinated fibers displayed robust Nav1.7 labeling at their nodes. In contrast to dorsal roots, nodes in ventral roots did not exhibit Nav1.7 immunolabeling (Figure 4).

**Figure 5:**
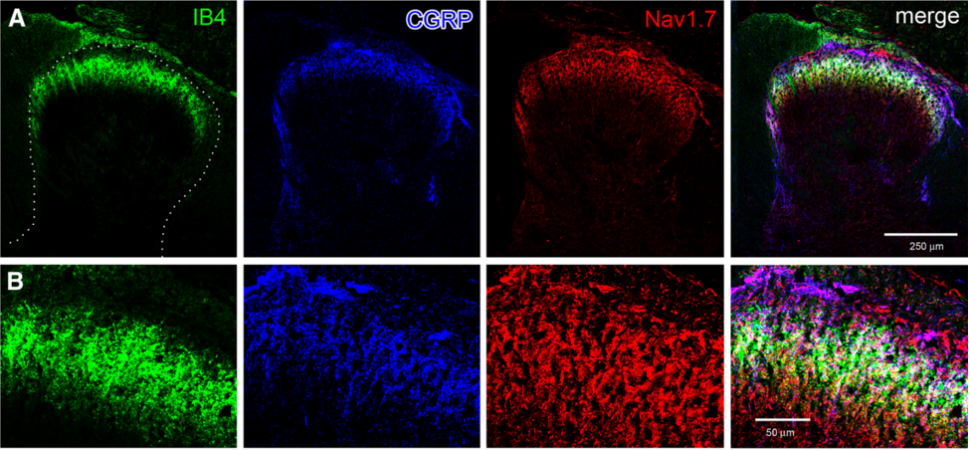
**Nav1.7 expression in spinal cord dorsal horn.** Sections of spinal cord were labeled for IB4 (green), CGRP (blue) and Nav1.7 (red). **A.** IB4 labeling is prominent in lamina IIi , while CGRP immunoreactivity is localized to lamina I and IIo. Robust Nav1.7 immunolabeling is present within lamina I and II, and exhibits co-localization with IB4 (yellow) and with CGRP (magenta). There is limited overlap of IB4 and CGRP in the superficial lamina. **B.** Increased magnification of IB4, CGRP and Nav1.7 labeling in superficial lamina of dorsal horn.

As demonstrated in Figure 1, cell bodies of IB4- and CGRP-positive DRG neurons exhibit robust Nav1.7 labeling. The central projections of IB4- and CGRP-positive neurons are targeted to differing lamina of the spinal cord dorsal horn, with IB4-positive terminating in lamina IIi and CGRP-positive terminating in lamina I and IIo [36]. In spinal cord sections triple-labeled with Nav1.7, IB4 and CGRP, prominent Nav1.7 immunoreactivity was present in superficial lamina of dorsal horns (Figure 5). The Nav1.7 immunolabeling was colocalized with IB4 staining in lamina IIi and with CGRP labeling in lamina I and IIo. 

**Figure 6:**
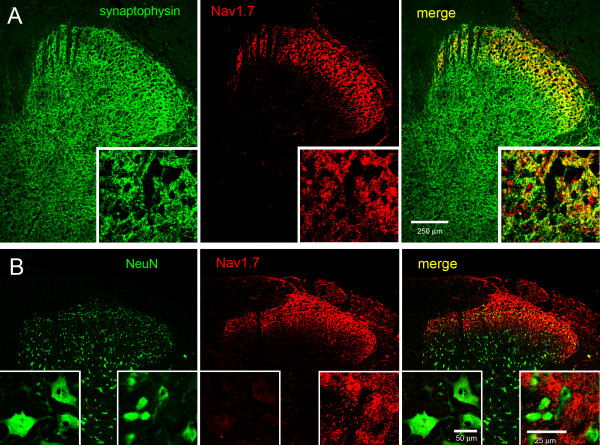
**Nav1.7 is expressed in pre-synaptic fibers in dorsal horn. A.** Synaptophysin (green), a marker of synapses, exhibits prominent labeling in the dorsal horn. Nav1.7 (red) and synaptophysin display extensive colocalization in the superfical layers of the dorsal horn. Inset. Increased magnification of superfical lamina of dorsal horn demonstrates colocalization of synaptophysin and Nav1.7. **B.** NeuN (green) immunolabels nuclei and cell bodies of neurons in dorsal horn. Post-synaptic neurons in the superficial lamina exhibit a lack Nav1.7 (red) labeling, which is localized in extracellular regions to the post-synaptic neurons. Right inset: increased magnification demonstrating lack of Nav1.7 immunoreactivity (red) within NeuN-labeled (green) dorsal horn neuronal cell bodies. Left inset: NeuN-labeled motor neurons (green) in ventral horn lack Nav1.7 immunolabeling (red).

The labeling pattern of Nav1.7 with IB4 and CGRP in the dorsal horn strongly suggests that Nav1.7 is localized within pre-synaptic central terminals of nociceptive DRG neurons. To address this suggestion, we co-labeled spinal cord sections with synaptophysin, a marker of synapses [39], and Nav1.7. As expected, substantial synaptophysin labeling was present within the dorsal horn, consistent with the formation of numerous synapses within this region. Laminas I and II of the dorsal horn exhibited a high degree of co-localization of Nav1.7 with synaptophysin (Figure 6), consistent with localization of Nav1.7 within pre-synaptic terminals. To determine whether the Nav1.7 immunoreactivity within superfical lamina of dorsal horn might reflect labeling of post-synaptic neurons, spinal cord sections were also labeled with NeuN, a marker of neuronal nuclei and to a lesser extent cytoplasm [40], and Nav1.7. An abundance of neurons were labeled with NeuN in lamina I and II of the dorsal horn (Figure 6). However, these NeuN-positive cells did not exhibit Nav1.7 immunolabeling, rather labeling was localized extracellular to these neurons. Notably, spinal cord ventral motor neurons did not exhibit Nav1.7 immunolabeling above background levels (Figure 6 inset) 

## Discussion

The present results demonstrate that Nav1.7 is robustly expressed in the somata of virtually all small diameter (<30 μm) DRG neurons, which predominantly give rise to nociceptive C- and Aδ-fibers [26,41]. In addition, Nav1.7 is expressed in peripheral and central terminal processes of these DRG neurons, with robust expression in the intraepidermal nerve fibers (IENF) within skin and the superficial lamina of spinal cord dorsal horn, the major site of synaptic connectivity between primary nociceptive and secondary sensory neurons. The preferential expression of Nav1.7 in small diameter DRG neurons and its localization at sites of nociceptive impulse genesis and synaptic transmission are consistent with major roles for this channel at multiple loci, extending from peripheral terminals in the skin to central axonal branches and terminals in the dorsal horn, in first-order neurons within peripheral pain pathways.

The Nav1.7 channel has recently garnered substantial interest in pain research, due to its association with human pain disorders, including inherited channelopathies, diabetic neuropathy, small fiber neuropathy, neuromas and inflammation. Loss-of-function mutations in Nav1.7 are associated with congenital insensitivity to pain (CIP) [7-9], while gain-of-function mutations in Nav1.7 are linked to the painful conditions of inherited erythromelalgia and paroxysmal extreme pain disorder (PEPD) [10,11,14,42]. In addition, Faber et al. [18] recently identified gain-of-function variants in Nav1.7 in nearly 30% of patients who met stringent criteria for idiopathic small fiber neuropathy, including reduced IENF and chronic pain. These studies provide a clear association between inherited alterations in Nav1.7 channel function and pain perception.

The activity of wild-type Nav1.7 channels is also implicated in chronic human pain syndromes. Injury to peripheral nerves can result in the formation of painful neuromas, tangles of proliferating connective tissue and blind-ending axons, which often exhibit spontaneous ectopic activity [43,44]. Nav1.7 has been shown to accumulate in the blind-ending axons of painful human neuromas [45-47]. Interestingly, the MAP kinase, ERK1/2, which phophorylates Nav1.7 and enhances its activation [48], also accumulates in painful human neuromas [47], and has recently been shown in experimental neuromas to co-localize within individual axons with Nav1.7 [49].

Animal studies provide additional support for a major contribution of Nav1.7 in nociception and chronic pain. Experimentally-induced diabetes results in mechanical allodynia and thermal hyperalgesia that is accompanied by an upregulation of Nav1.7 in DRG neurons [50-52]. Significantly, continuous δ-opioid receptor activation via HSV-infection of DRG with a proenkephalin-expressing vector attenuated responses to noxious thermal and mechanical stimuli and the increased Nav1.7 expression in diabetic rats [53]. Nav1.7 has also been shown to play an important role in inflammatory pain. Experimental models of inflammation have been shown to induce upregulation of Nav1.7 [54-56]. HSV-delivered antisense sequence to Nav1.7 in hindpaws of mice injected with Freund’s adjuvant prevented an increase in Nav1.7 expression that is accompanied by decreased hypersensitivity compared to control mice [57].

A major role for Nav1.7 in inflammatory pain is supported by knock-out studies. Deletion of Nav1.7 in Nav1.8-expressing DRG neurons, which are principally nociceptive, greatly attenuated or eliminated behavioral responses to a range of inflammatory agents [58]. Interestingly, mechanical and thermal responses to noxious stimuli were not altered in these knock-out mice. However, it was recently shown that ablating Nav1.7 in all sensory neurons within DRG in Advillin-Cre/1.7 loxP mice abolished mechanical, inflammatory and thermal, but not neuropathic, pain responses [59]. Neuropathic pain was eliminated only when Nav1.7 was knocked out in DRG sensory neurons and sympathetic neurons. Interestingly, in a burn injury model utilizing conditional Nav1.7 knockout mice, Shields et al. [60] demonstrated that Nav1.7 selectively contributed to burn-induced hypersensitivity to heat but not mechanical stimuli. In addition, whole cell patch clamp studies showed an increase in TTX-sensitive current density and hyperpolarizing shift in steady-state activation in DRG neurons following burn injury in WT mice that was absent in Nav1.7 knockout mice [60], consistent with a contribution of Nav1.7 channels to increased excitability of DRG neurons following burn injury. In the aggregate, these animals studies convincingly demonstrate an important contribution of Nav1.7 to nociception and pain syndromes.

The Nav1.7 sodium channel exhibits fast-activation and fast-inactivation, similar to the other tetrodotoxin-sensitive (TTX-S) channels, Nav1.1, Nav1.2, Nav1.3, and Nav1.6, expressed in nervous tissue [5,20]. However, Nav1.7 displays unique properties that poise it to play a critical role in affecting the excitability of neurons that express it. Nav1.7 is distinguished from other TTX-S channels by a slow recovery from fast inactivation (slow repriming) [21,22]. In addition, Nav1.7 has a slow closed-state inactivation that yields a sodium current (ramp current) in response to small, slow depolarizations. These properties position Nav1.7 channels to amplify generator potentials and thus act as a threshold channel for setting the sensitivity of action potential electrogenesis [23]. In nociceptive neurons, increased activity or density of Nav1.7 channels, as in gain-of-function mutations and inflammation, respectively, would be expected to lower the threshold for firing and amplify the response to stimuli, likely leading to enhanced pain perception.

Nav1.6 is the predominant sodium channel isoform aggregated at nodes of Ranvier in adult tissue [61]. However, our results demonstrate robust Nav1.7 expression at nodes of Ranvier in a subpopulation of small diameter (Aδ) myelinated fibers in sciatic nerve and dorsal root. The expression of Nav1.7 at these nodes is coincident with the expression of Nav1.6, as <90% of the Nav1.7-positive nodes co-expressed detectable Nav1.6 (data not shown). While it cannot be unambiquously determined that fibers with the Nav1.7-positive nodes are continuous peripheral and central projections of a subset of DRG neurons, sciatic nerve and dorsal root have nearly equal percentages (36 vs. 34%, respectively) of these fibers with nodal Nav1.7 labeling, suggesting the presence of a subset of fibers that express Nav1.7 at nodes from the skin to dorsal horn. At this time it cannot be determined whether expression of Nav1.7 at nodes in a subset of Aδ-fibers is associated with a specific function. In this regard, it has been reported that 40% of Aδ-fibers are only mechanoresponsive [62], which is similar to the percentage of Aδ-fibers expressing Nav1.7 at nodes in sciatic nerve and dorsal root . It is not clear how co-expression of Nav1.7 and Nav1.6 may effect pain signal transmission along thse fibers, but it may provide a high safety factor for the conduction of noxious mechical stimuli.

We demonstrated the co-expression of Nav1.7 with CGRP and IB4 in lamina I/IIo and IIi, respectively, of spinal cord dorsal horn. We further demonstrated co-localization of Nav1.7 with synaptophysin, a marker of pre-synaptic terminals, and a lack of Nav1.7 labeling in NeuN labeled post-synaptic neurons in the superficial layers of dorsal horn. These results support the observations of Minett et al. [59] in which the stimulated release of Substance P in dorsal horn was significantly attenuated in Nav1.7 null DRG neurons compared to WT mice. Taken together, these results provide evidence for a contribution of Nav1.7 in the regulation of neurotransmitter release in nociceptive fibers. Interestingly, Nav1.7 is the predominant sodium channel expressed in rodent olfactory sensory neurons, with Nav1.7 accumulation extending to the presynaptic termini in the glomeruli of the olfactory bulb [28], and a critical role for Nav1.7 in the regulation of synaptic transmission by the olfactory sensory neurons has been convincingly demonstrated [29].

## Conclusions

In summary, our results demonstrate that Nav1.7 is expressed by nociceptive DRG neurons along their entire trajectory, with expression extending from the peripheral terminals of IENF in the skin to preterminal projections of these axons with the dorsal horn, and to central synaptic terminals in the spinal cord. The expression of Nav1.7 at central and peripheral terminals, as well as along the peripherally- and centrally-directed trunk of nociceptive fibers, suggests functional contributions of Nav1.7 at multiple foci within pain pathways, extending from peripheral axons within the skin to central preterminal axons and axon terminals in the dorsal horn. These observations may have important implications for development of pain pharmacotherapy, where target engagement in the right compartment is essential.

## Methods

### Animal care

Sprague–Dawley male rats (adult, 225–250 gm, Harlan, Indianapolis, IN) were housed under a 12 hr light/dark cycle in a pathogen-free area with *ad libitum* access to water and food. The experimental procedures were approved by the VA Connecticut Healthcare System Institutional Animal Care and Use Committee, in accordance with NIH guidelines.

#### Immunocytochemistry

Rats were deeply anesthetized with ketamine/xylazine (80/5 mg/kg, i.p.) and transcardially perfused with 0.01 M PBS (pH 7.4) followed by ice-cold 4% paraformaldehyde in 0.14 M Sorensen’s phosphate buffer (pH 7.4). Tissues (sciatic nerve, L4 and L5 dorsal root ganglia, dorsal roots, ventral roots, and spinal cord) were removed, immersion-fixed for an additional 20 min (total fixation time 30 min) and cryoprotected with 30% (w/v) sucrose in PBS overnight at 4°C. Hindpaw glabrous skin was immersion-fixed in Zamboni’s fixative for 8 hours at 4°C, which yielded more robust labeling of intraepidermal nerve fibers than paraformaldehyde fixation, prior to cryoprotection. Ten-μm thick cryosections were mounted on slides (Fisher, Pittsburgh, PA) and processed for detection of Na_v_1.7 protein and cell-specific markers as described previously [54]. In brief, sections were incubated in the following (1) blocking solution (PBS containing 3% cold water fish skin gelatin, 3% normal donkey serum, 2% BSA, 0.1% Triton X-100, and 0.02% sodium azide) for 15 min at room temperature; (2) primary antibodies [rabbit anti-Nav1.7 (1:250, Y083 [47]; mouse anti-peripherin (1:1000, Abcam, Cambridge, MA); chicken anti-neurofilament 200 (1:1000, Aves Lab, Tigard, OR), IB4-Alexa Fluor 488 (1:100, Invitrogen, Carlsbad, CA), sheep anti-calcitonin gene-related protein (1:100, Abcam); mouse anti-PGP9.5 (1:2000, Encor Biotechnology, Gainsville, FL), guinea pig anti-caspr (1:2000, 085 [13]) and mouse anti-synaptophysin (1:50, GeneTex, Irvine, CA)] in blocking solution for 24–48 hours at 4°C; (3) PBS, 6 × 5 min each; (4) appropriate secondary antibodies in blocking solution for 12–24 h at 4°C; (5) PBS, 6 × 5 min each. Control experiments were performed without inclusion of primary antibodies, which yielded only background levels of fluorescence (data not shown). Tissue sections were examined with a Nikon C1 confocal microscope (Nikon USA, Melville, NY) using a 20x objective and operating with frame lambda (sequential) mode and saturation indicator to prevent possible bleed-through between 488, 549 and 633 nm channels.

#### Quantitative analysis

Images of dorsal roots (3 sections each for n=4 rats), sciatic nerves (3 sections each for n=4 rats) and DRG (3 sections each for n=5 rats) were acquired, yielding 12, 12, and 15 separate images of dorsal root, sciatic nerve and DRG tissue, respectively, for quantification.

For determination of co-localization of Nav1.7, peripherin, and neurofilament in sciatic nerve and dorsal root, a line was placed on the images orthogonal to the axis of the fibers, which extended from edge to edge of the tissue (~350-500 μm). Nav1.7 (red)-, peripherin (green)- and NF (blue)-positive fibers (at least 10 μm in length) that intersected the line were counted separately and merged (i.e. Nav1.7 and peripherin = yellow). Percentage of peripherin- or neurofilament-positive fibers expressing Nav1.7 was calculated as total number of peripherin- or neurofilament-positive fibers co-localized with Nav1.7 (i.e. yellow or violet, respectively) divided by the total number of peripherin or neurofilament-positive fibers (i.e. green or blue).

For determination of Nav1.7 immunolabeling at nodes of Ranvier, sections of sciatic nerve and dorsal root were reacted with antibodies to Nav1.7 and caspr, which is a marker of paranodes [37]. Images were acquired of every small diameter (<1 μm) node in the section, and the percentage of Nav1.7-positive nodes calculated.

To determine the number of DRG neurons expressing Nav1.7 (red), IB4 (green),CGRP (blue), peripherin (green) and neurofilament (blue) in triple-labeled sections (i.e. Nav1.7, IB4 and CGRP; Nav1.7, peripherin and neurofilament), the number of positive neurons for each channel was assessed. The numbers of neurons exhibiting co-localization of Nav1.7/IB4, Nav1.7/CGRP, Nav1.7/peripherin and Nav1.7/neurofilament were then counted and the percentage of neurons displaying colocalization was calculated.

## Abbreviations

Caspr: Contactin associated protein; CGRP: Calcitonin gene-related protein; CIP: Chronic insensitivity to pain; DRG: Dorsal root ganglia; ERK1/2: Extracellular signal-regulated kinase 1/2; HSV: Herpes simplex virus; IB4: Isolectin B4; IENF: Intraepidermal nerve fiber; MAP kinase: Mitogen-activated protein kinase; NeuN: Neuronal nuclei; PEPD: Paroxysmal extreme pain syndrome; TTX-S: Tetrodotoxin-sensitive; WT: Wild-type.

## Competing interests

The authors declare that they have no competing interests.

## Authors’ contributions

JAB designed immunocytochemical experiments, acquired, analyzed and interpreted data, and participated in writing the manuscript. NF performed immunocytochemical experiments and acquired, analyzed and interpreted data. SDH and SGW participated in design of experiments and edited the manuscript. All authors read and approved the final manuscript.
